# How changes in pubertal status and chronological age shape individual depressive symptoms by sex: testing a pathoplasty model

**DOI:** 10.1007/s00787-026-03065-9

**Published:** 2026-05-18

**Authors:** Pascal Schlechter, Sharon A. S. Neufeld, Lindsey Stewart, Paul O. Wilkinson, Thomas M. Olino

**Affiliations:** 1 Department of Psychology, University of Münster, Münster, Germany; 2 Department of Psychiatry, University of Cambridge, Cambridge, England; 3 Department of Psychology & Neuroscience, Temple University, Philadelphia, United States

**Keywords:** Depression, Puberty, Pathoplasty model, Tanner stages, Sex

## Abstract

Depressive symptoms increase during puberty and females experience higher rates of depression compared to males. However, it is unclear whether the increase in depressive severity is due to general increases in all symptoms, or whether there are specific symptoms that are more prominently influenced by pubertal development. Therefore, we examined whether pubertal status and chronological age exert pathoplastic effects (i.e., differential influences on disorder expression) on depressive symptoms. To isolate pathoplastic effects, we controlled for overall depression severity. Data came from 7,362 participants spanning ages 10–18 in the Avon Longitudinal Study of Parents and Children. Depression was assessed via the Short Mood and Feelings Questionnaire. Pubertal status was measured through self- and parent-reports of pubic hair, breast, and gonadal development, and voice changes in male participants. Pubertal changes correlated with overall depression severity in females, but not in males. Certain symptoms were more prominently influenced by pubertal indicators and chronological age, supporting pathoplastic effects. In females, within-person breast development was associated with increases in the symptoms *I hated myself* and *nobody really loved me* and decreases in the symptom *felt lonely*. Pubic hair development was associated with higher levels of *miserable/unhappy* and *I hated myself*. In males, within-person testes development was associated with increases of the symptom *felt tired*. Voice changes were associated with increases in the symptom *felt lonely*. Chronological age was associated with multiple symptoms, beyond the effects of puberty. Pubertal changes and age independently shape depressive symptoms in different ways by sex.

**Supplementary Information:**

The online version contains supplementary material available at https://doi.org/10.1007/s00787-026-03065-9.

From the onset of puberty in early adolescence, females experience higher rates of depression compared to males [1, 2]. Females also tend to begin pubertal development earlier than males [3]. Gender/sex-related differences in depression prevalence and presentation may reflect the interplay between hormonal changes on the brain (indexed by biological sex characteristics) and gender-specific social changes during puberty [4–6]. However, it remains unclear whether the increase in depression severity in females is due to general increases in all symptoms, or whether there are specific symptoms that are more prominently influenced by pubertal development. The pathoplasty model attempts to explain observed heterogeneity in the expression of symptoms by considering factors which influence the clinical manifestation of depression [7, 8]. However, this model has not been applied to test whether pubertal indicators yield differential effects on depressive symptoms. In this study, we examined how within-person changes in pubertal markers (i.e., Tanner stages of pubic hair, breast, voice, and gonadal development) influence individual depressive symptoms while controlling for overall depression severity. We also accounted for chronological age to distinguish the independent effects of age and pubertal stage, given they are strongly correlated, but may reflect different processes influencing symptoms [4, 9].

While there are several ways of operationalizing pubertal development, including timing, tempo, and pubertal synchrony (reviewed in [10, 11]), Tanner staging provides a standardized assessment of *pubertal status* based on visible physical changes in the body [12]. In the present work, pubertal development is indicated by within-person changes in Tanner stages: breast development in females, testes development in males, and pubic hair growth in both sexes. These hallmark features of puberty signify the activation of the hypothalamic-pituitary-gonadal and hypothalamic-pituitary-adrenal axes, resulting in the production of sex hormones, including estrogen and testosterone [13]. These hormonal fluctuations have profound effects on brain development, behavior, and emotional regulation [3, 5]. Hormonal changes differ between males and females [13], and the role of specific hormones within each sex differs across puberty [14]. In females, increases in estrogen during breast development may contribute to a higher vulnerability to depression [11]. Fluctuations in estrogen during the menstrual cycle have been associated with affective variability and increased risk of depression [15, 16]. In males, the surge in testosterone during testicular development may be protective against depression [17]. Dehydroepiandrosterone (DHEA) and its sulfated form (DHEA-S), which increase during adrenarche and drive the development of body odor, acne, and pubic/underarm hair, have also been implicated in mood regulation [18]. In adolescent populations, DHEA/DHEA-S levels and the cortisol–DHEA ratio have been associated with depressive symptoms and disorder course, although findings are heterogeneous and suggest that effects are likely developmentally sensitive and moderated by pubertal status and sex [19]. Tanner stages are strongly associated with hormonal markers of maturation, as well as physical examinations [6]. Accordingly, we focus on these indicators to examine pathoplastic influences of *changes in pubertal status* on the expression of depressive symptoms [20]. In addition, we consider voice changes in males as a socially salient indicator of pubertal status [21].

According to a recent systematic review, advanced *pubertal stage* is associated with depression among females, while among males this association is typically not statistically significant [11], for research on timing effects, see [22, 23]. More specifically, early breast development has been associated with increased risk of depression in adolescent females [24]. Early menarche (< 11.5 years) was linked to heightened levels of depressive symptoms during early to middle adolescence [25, 26], but not in later adolescence and young adulthood [27, 28]. During mid-adolescence, pubertal status with respect to breast stage, rather than timing of menarche, has been related to depressive symptoms [29, 30].

While previous research has provided valuable insight into the association of pubertal status and depression, it has focused on overall depressive severity [29] or disorder onset [30], neglecting potential pathoplastic effects of pubertal status on individual depressive symptoms. Depression is a heterogeneous construct, and individuals with similar levels of overall severity may differ substantially in their symptom profiles [31]. As such, examining associations at the level of individual symptoms allows for a more precise understanding of how developmental processes shape the manifestation of depression. In the pathoplasty model, researchers examine how specific variables impact individual symptoms over time while also controlling for the overall severity of depression, enabling a nuanced exploration of how a predictor influences the manifestation of the disorder. This approach enables the identification of factors that influence *how depression is expressed*, rather than simply *whether it increases in severity*. This distinction is critical, as different symptom domains (e.g., self-evaluative, social, somatic) may reflect distinct underlying mechanisms and developmental pathways [31]. Consequently, focusing on individual symptoms may provide more specific insight into the processes linking pubertal development and depression and help guide future research and tailored treatments targeting these mechanisms. While investigations of the pathoplasty model of depression have considered personality [8, 32], episode number and age of onset [33], inflammation [34], and life events [35] puberty has not yet been tested as a predictor. Importantly, pathoplastic effects of pubertal status on individual depressive symptoms may reveal sex-specific routes into depression. Specifically, females with early breast development might be more susceptible to low mood or not enjoying daily activities [11]. In addition, puberty may affect the expression of cognitive symptoms of worthlessness based on sexual objectification by peers [1]. Males may be more likely to experience restlessness during puberty [17], which may contribute to higher levels of agitation and impulsivity, potentially leading to concentration problems.

With rare exceptions ([36, 37] – only in female individuals), most studies have solely investigated between-person differences to investigate the relationship between depression and pubertal status. However, to capture changes in pubertal status within individuals and how these changes affect depressive symptoms, within-person studies of change over time are needed. In the context of studying changes in pubertal status and their impact on depressive symptoms, within-person analysis examines how pubertal development unfolds within each individual and how this is associated with depressive symptoms over time. Neither of the prior two studies employing within-person methods have done so using a pathoplasty model.

The present study examines how within-person changes in pubertal status influence the presentation of individual depressive symptoms, while controlling for overall depression severity. Employing multiple waves of adolescents ages 10 to 18 from a birth cohort, Avon Longitudinal Study of Parents and Children (ALSPAC [38]), allows disaggregation of between- and within-person effects. To separate pubertal status from chronological age and other developmental effects [4], our analyses controlled for chronological age. This way, we aimed to isolate the effects of change in pubertal stage within an individual over time, independent of the effects of age. This discerns whether depressive symptoms are linked to the progression of puberty itself, not just the effects of time. We examined these effects separately for males and females to discern the distinct impacts of change by age across the Tanner stages for pubic hair, breast, and gonadal development on depressive symptoms. We also investigated voice changes in males, as a more externally-apparent indicator of development than gonadal development. Our work thus extends prior analyses of this cohort on associations of pubertal status and age on depressive symptoms [28, 29] by (1) considering individual depressive symptoms as the outcome, (2) considering within-person effects with (3) reported pubertal stage preceding symptoms, and (4) including males.

## Methods

### Participants and design

ALSPAC is a population-based study conducted in South-West England, with recruitment occurring from 1st April 1991 until 31st December 1992. Expectant mothers and their children (*N* = 14,541) were assessed throughout their life [38, 39]; 13,988 children were alive at age 1. When the oldest children had reached age 7, attempts were made to boost the sample, resulting in a final sample of 14,901 children alive at age 1. These individuals were the focus of data collection from the age 7 onwards. Compared to the general UK population, ALSPAC participants were more likely to be of White ethnicity, had higher levels of education, and were less likely to receive free school meals [38]. Table 1 shows demographic characteristics of the analyzed sample (*n* = 7362). The study website contains details of available data (http://www.bristol.ac.uk/alspac/researchers/our-data). The ALSPAC Law and Ethics Committee and Local Research Ethic Committee gave ethical approval for the study. Informed consent for the use of data collected via questionnaires and clinics was obtained from participants following the recommendations of the ALSPAC Ethics and Law Committee at the time. Full cohort profile descriptions are available [38].

**Table 1:** Demographic characteristics and descriptive statistics of SMFQ and Tanner Stages for each wave. Note. Number in brackets refer to percentages or standard deviations

	Wave 1 (*N* = 7362)	Wave 2 (*N* = 6716)	Wave 3 (*N* = 6019)	Wave 4 (*N* = 4997)	Wave 5 (*N* = 4497)
Age at SMFQ	10.65 (0.26)	12.81(0.23)	13.84 (0.21)	16.68 (0.24)	17.84 (0.40)
Age at Tanner Stage	9.70 (0.14)	11.80(0.14)	13.10 (0.15)	15.50 (0.29)	17.01 (0.09)
Sex					
Male	3621 (49%)	3281 (49%)	2963 (49%)	2019 (40%)	1905 (42%)
Female	3731 (51%)	3421 (51%)	3075 (51%)	2968 (59%)	2587 (58%)
SMFQ	4.04 (3.51)	3.97 (3.86)	4.92 (4.49)	5.91 (5.64)	6.59 (5.25)
Breasts	1.44 (0.63)	2.61 (0.99)	3.61 (0.97)	4.24 (0.66)	4.55 (0.58)
Testes	2.16 (0.91)	2.86 (1.15)	3.59 (1.19)	4.19 (0.73)	4.66 (0.60)
Pubic Hair (f)	1.23 (0.52)	2.47 (1.29)	3.82 (1.20)	4.50 (0.61)	4.74 (0.54)
Pubic Hair (m)	1.18 (0.40)	2.03 (1.19)	3.19 (1.37)	4.35 (0.65)	4.77 (0.49)
Voice Change	1.04 (0.20)	1.16 (0.40)	1.59 (0.72)	2.38 (0.87)	2.45 (0.71)

Number in brackets refer to percentages or standard deviations. Continuous data are presented as mean (standard deviation)

*SMFQ* Short Mood and Feelings Questionnaire, *f* female, *m* male

### Measures

#### **Short Mood and Feelings Questionnaire**

The Short Mood and Feelings Questionnaire (SMFQ) was self-reported to assess depressive symptoms during adolescence. The SMFQ was assessed in the first five study waves spanning ages 11 to 18. The SMFQ consists of 13 items, with the response categories “*not true*” (0), “*sometimes*” (1), and “*true*” (2), resulting in scores from 0 to 26. The SMFQ is a reliable and valid screening instrument designed for children and adolescents [40] and has displayed longitudinal measurement invariance across ages 11–17 and ages 14–26 in ALSPAC [41]. Cross-sectional partial measurement invariance was found across sex for all waves included in the present analysis (ibid.). Cronbach’s alpha ranged from 0.80 (wave 1) to 0.91 (wave 5).

#### **Tanner Stages**

We used data from five questionnaires to evaluate pubertal status, which were filled out by the primary caregiver with or without their child’s input [42]. Our analyses structured the data so that pubertal status preceded each assessment of depressive symptoms (Table 1). Tanner stages were assessed at mean ages 9.7, 11.8, 13.1, 15.5, and 17 for the five waves. Parents were involved in Tanner reporting for the first three assessments but for the last two assessments, the majority of participants (over 95% for both sexes) completed them without parental assistance. For the second, third, and fourth assessments, females received more parental support in completing the Tanner scales than males (all - values > 51.14 and ps < 0.001). Prior work has shown high correspondence between maternal assessment and clinical evaluation both in males and females ages 9–18 (Cohens Kappa 0.82–88; [43–45]). Yet, more recent work shows that older children provide more accurate reports compared to their mothers (female individuals aged ≥ 11 years compared to younger females; [46]). The questionnaires incorporated both visual representations (schematic drawings) and verbal descriptions of secondary sexual characteristics, such as breasts and pubic hair, based on the Tanner staging system [12, 47]. The Tanner scale consists of five stages, ranging from stage 1 to stage 5. Higher stages indicate more advanced development of secondary sex characteristics, while lower stages indicate less mature or prepubertal development. We used four different pubertal indicators based on Tanner stages, two for each sex. For females, we used pubic hair development and breast development. For males, we used pubic hair development and developmental stage of testes, scrotum, and penis. We also examined voice changes in males (assessed as “no change”, “occasionally a little lower”, and “changed totally”). Inconsistent ratings (i.e., regression in Tanner stages) ranged from less than 1% (in most assessments for both sexes) to 6.9% for females (pubic hair development at ages 15.5 vs. 17) and 8.5% for males (testes development at ages 9.7 vs. 11.8). There may be greater measurement error in assessing testes development, indicated by previous inconsistent reporting, with Tanner stages deviating from age expectations [42]. Additionally, the subjective nature of assessing testes size and pubic hair development may contribute to these variations. Since no other inconsistencies were detected when using the careless package in R [48], these participants were retained in our analyses. Sex was reported based on biological sex (sex assigned at birth in this birth cohort study, we therefore use the terms male and female).

### Data analysis

Data were analyzed with Mplus 8.10 and facilitated using the *MplusAutomation* package in R [49]. Data were treated as missing at random (MAR) in line with former reports on SMFQ in ALSPAC, which identified being male, having mothers with poorer educational attainment, a younger mother, and higher depression at age 11 as factors associated with missingness [41]. The highest level of missingness compared to SMFQ wave 1 (the first time SMFQ was assessed) was 39% (4497/7362) at SMFQ wave 5. For the pubertal indicators, the percentage of missing data among the 7,362 participants ranged from 22.9% (for pubic hair development in girls at age 9.7) to 61.4% (for testes development in boys at age 17). Across the 10 puberty variables (two variables per sex measured at 5 waves), missing data were more common in males than female for 8 out of 10 cases (all *p*s < 0.01), in line with former ALSPAC reports [42].

**Fig. 1:**
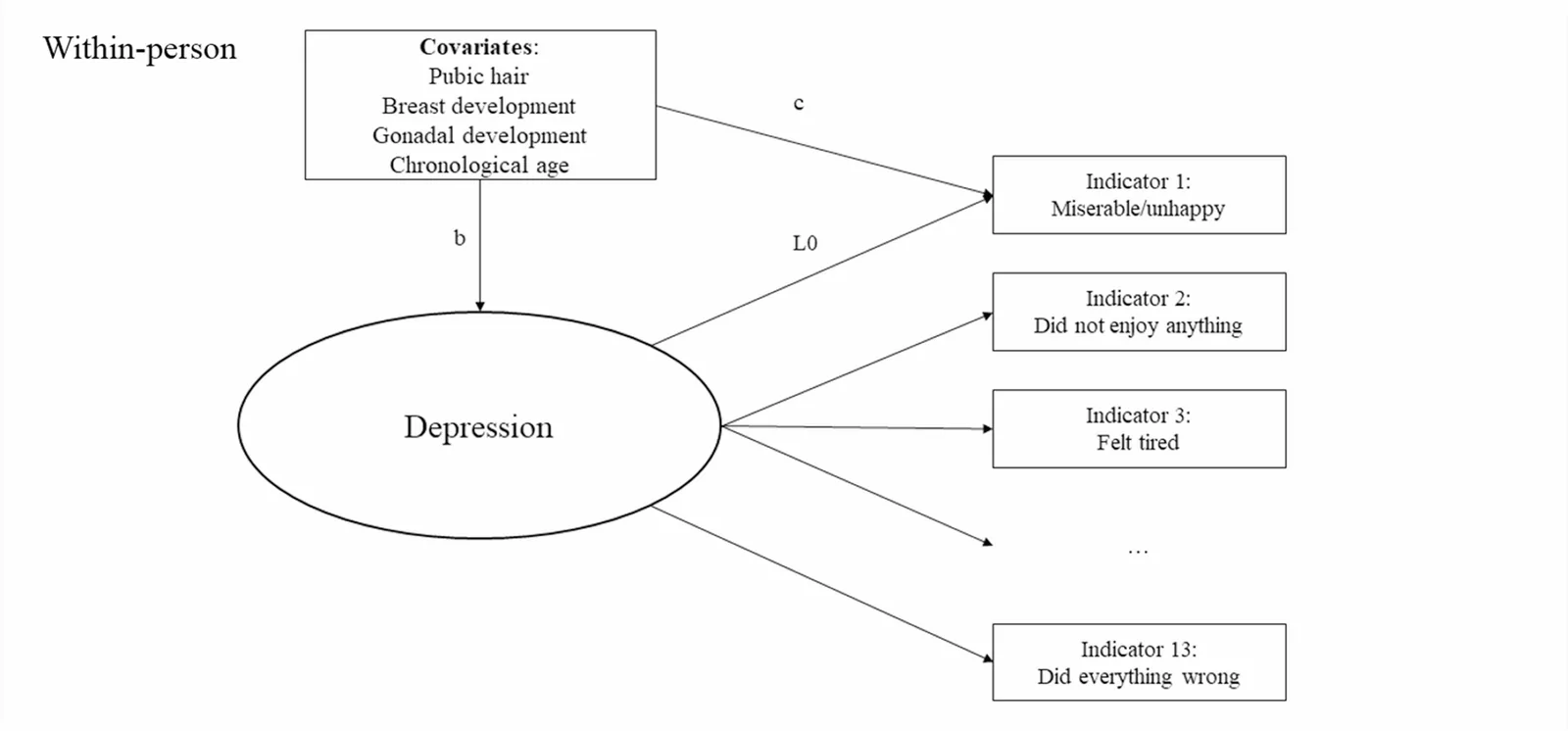
Simplified illustration of the multilevel structural equation model for depressive symptoms. Note. The thirteen indicators/symptoms of depression as measured by the SMFQ are all captured by an underlying construct coined depression. For each individual symptom, path L0 presents the loading of the individual indicator on the latent factor. Path b presents the impact of the covariates on the latent depression factor via a regression coefficient. Path c presents the impact of the covariates on the individual indicator via a regression coefficients. Models are estimated separately for each pubertal factor (adjusted by age) and separately for each indicator

Previous work indicates that SMFQ can be represented by a one-factor model across development in ALSPAC [41]. Therefore, we used a one-factor model and examined the effects of pubertal status and chronological age on each individual symptom while simultaneously accounting for the latent depression construct [34]. Controlling for overall depressive severity is a prerequisite for testing the pathoplasty hypothesis. Pathoplastic effects concern factors that influence the expression of a disorder, independent of overall severity. Accordingly, we included a latent depression factor in all models to account for general symptom severity. This allows the estimated effects of pubertal status and age on individual symptoms to be interpreted as symptom-specific deviations from overall depression. We estimated the influence of pubertal status on specific symptoms using multi-level structural equation modeling with robust maximum likelihood estimation with numerical integration due to the categorical indicator variables. This is a full-information estimator, so all available data points are used based on the MAR assumption [50]. Analyses examined the effect of each time-varying predictor (pubertal indicators) on each of the thirteen individual depressive symptoms. This led to a total of 2 (pubertal indicators for females) x 3 (pubertal indicators for males) x 13 (depressive symptoms) = 65 models, or 26 models for females and 39 models for males (the number of models tested is similar for other pathoplasty models; [32]). Pubertal status was coded such that Tanner stage 1 had a value of 0 for interpretation of the coefficients. In all models, chronological age (with the youngest age category set to zero) was included as an additional time-varying covariate on the individual symptoms. This way, our models calculate the effect of pubertal status as if all participants were the same age, removing any effects common to both age and pubertal status. Figure 1 presents a schematic representation of the models. Results are based on ordinal regression models and yield odds ratios (ORs). Given the large number of statistical tests, we used Benjamini-Hochberg correction for our p-values [51] and placed 99% confidence intervals (CI) around the estimates.

## Results

Table 1 presents descriptive statistics of the SMFQ and Tanner stages over time. Across models in females, higher Tanner stages of breast development (*bmean* = 0.07; *SDb* = 0.003, *p* < .01) and pubic hair development (*bmean* = 0.09, *SDb* = 0.003, *p* < .001) were independently associated with higher levels of latent depression (results were similar across the models; the mean coefficient is presented). In male models, pubertal indicators were not associated with latent depression (*bmean* = 0.01; *SDb* = 0.005, *p* = .61). Across models in both sexes, higher chronological age was associated with higher levels of latent depression (females: *bmean* = 0.19; *SDb* = 0.01, *p* < .001; males: *bmean* = 0.06; *SDb* = 0.01 *p* < .01). Table 2 shows the effects of pubertal indicators and age on depressive symptoms while controlling for latent depression. In both sexes, age and pubertal indicators correlated strongly, *r*s = 0.67-0.81, all *p*s < 0.001. Supplementary Tables 1 and 2 present the single effects of pubertal status and age. In these models, the direction of effects for the same parameter (e.g., pubertal level predicting symptom; age predicting symptom) were the same. We focus the remaining presentation of the results on the models with pubertal markers and age in the same model to discern their unique contributions.

**Table 2:** Effects of the puberty indicators and chronological age on the depressive symptoms while controlling for the latent depression trait

Symptom	Breasts	Age (f)	Pubic hair (f)	Age (f)	Testes	Age (m)	Pubichair (m)	Age (m)	Voice (m)	Age (m)
1.	1.06(0.98-1.14)	1.02(0.94-1.11)	1.07(1-1.15)*	1.00(0.93-1.08)	0.99(0.92-1.06)	1.11(1.04-1.18)***	1.01(0.94-1.08)	1.09(1-1.18)	1.07(0.95-1.21)	1.07(1.00-1.15)*
2.	1.00(0.9-1.09)	0.99(0.9-1.09)	0.95(0.88-1.04)	1.03(0.94-1.13)	1.03(0.94-1.13)	1.03(0.94-1.12)	1.03(0.93-1.13)	1.04(0.93-1.16)	0.88(0.76-1.03)	1.09 (1-1.19)*
3.	0.98(0.91-1.04)	1.25(1.16-1.34)***	0.98(0.92-1.04)	1.25(1.17-1.34)***	1.08(1.01-1.15)*	1.11(1.05-1.18)***	1.02(0.95-1.09)	1.13(1.05-1.23)***	0.95(0.85-1.06)	1.2(1.13-1.29)***
4.	0.95(0.89-1.02)	1.01(0.94-1.09)	0.98(0.92-1.03)	0.99(0.92-1.06)	1.01(0.95-1.07)	0.99(0.93-1.05)	1.00(0.94-1.07)	0.99(0.91-1.06)	1.04(0.93-1.15)	0.98(0.92-1.04)
5.	1.01(0.9-1.13)	0.89(0.79-1)*	1.03(0.94-1.13)	0.87(0.78-0.97)**	0.97(0.87-1.1)	0.92(0.82-1.03)	0.99(0.87-1.12)	0.92(0.8-1.07)	0.96(0.78-1.17)	0.94(0.84-1.06)
6.	0.97(0.89-1.05)	1.10(1.02-1.2)**	0.97(0.91-1.05)	1.10(1.02-1.2)**	0.91(0.8-1.03)	0.81(0.72-0.92)***	0.89(0.78-1.02)	0.85(0.73-1)*	0.91(0.73-1.13)	0.8(0.71-0.9)***
7.	0.98(0.92-1.05)	1.07(0.99-1.15)	0.99(0.93-1.05)	1.06(0.98-1.14)	1.03(0.97-1.11)	1.04(0.98-1.12)	1.00(0.93-1.07)	1.07(0.98-1.16)	1.06(0.94-1.19)	1.04(0.97-1.11)
8.	1.11(1-1.23)*	0.77(0.69-0.86)***	1.10(1-1.21)*	0.77(0.69-0.86)***	0.97(0.85-1.11)	0.9(0.79-1.01)	0.99(0.85-1.14)	0.88(0.75-1.04)	0.98(0.79-1.22)	0.89(0.78-1.01)
9.	1.06(0.96-1.17)	1.22(1.11-1.35)***	1.08(0.98-1.18)	1.20(1.09-1.33)***	1.03(0.93-1.13)	1.10(1-1.2)	1.04(0.93-1.16)	1.08(0.95-1.22)	1(0.85-1.17)	1.12(1.02-1.23)***
10.	0.90 (0.83-0.98)**	1.04 (0.96-1.13)	0.96 (0.9-1.03)	1.00 (0.92-1.08)	0.99 (0.9-1.08)	1.15 (1.05-1.25)***	1.02 (0.93-1.12)	1.12 (1-1.24)	1.19 (1.02-1.39)***	1.06 (0.97-1.16)
11.	1.13(1.01-1.26)*	0.82(0.74-0.92)***	1.03(0.94-1.14)	0.88(0.79-0.98)**	1.01(0.88-1.15)	1.06(0.94-1.21)	1.01(0.87-1.16)	1.08(0.92-1.26)	1.1(0.88-1.39)	1.03(0.9-1.18)
12.	0.97(0.89-1.06)	0.85(0.77-0.93)***	0.94(0.87-1.01)	0.88(0.8-0.95)***	0.94(0.85-1.03)	0.86(0.79-0.95)***	0.91(0.82-1.02)	0.90(0.8-1.01)	0.86(0.73-1.01)	0.88(0.8-0.96)
13.	0.97(0.87-1.07)	1.00(0.9-1.11)	0.93(0.85-1.02)	1.05(0.95-1.16)	1.08(0.96-1.2)	0.88(0.8-0.98)**	1.08(0.96-1.21)	0.86(0.75-0.98)*	0.87(0.72-1.04)	0.96(0.87-1.06)

Results are based on ordinal regression models and present odds ratios

Note that these are Benjamini-Hochberg corrected p-values

*f* female, *m* male

1. Miserable/unhappy, 2. Did not enjoy anything, 3. Felt tired, 4. Restless, 5. I was no good anymore, 6. Cried a lot, 7. Hard to concentrate, 8. I hated myself, 9. I was a bad person, 10. Felt lonely, 11. Nobody really loved me, 12. Not as good as other kids, 13. Did everything wrong

### Pubic hair

In females, pubic hair development was associated with higher subsequent levels of *miserable/unhappy* and *I hated myself.* In males, pubic hair development was not associated with any symptoms.

### Breast and testes development

In females, breast development was associated with within-person increases of the symptoms *I hated myself* and *nobody really loved me* and with lower levels of *felt lonely.* There was only overlap in one symptom (*I hated myself*) between pubic hair development and breast development in female individuals. In males, testes development was associated with higher levels of *felt tired*.

### Voice changes

In the models including voice changes and age, most effects were explained by age. Yet voice changes positively predicted increased levels of the symptom *felt lonely.*

### Age effects

In females, age was associated with higher levels of *felt tired*, *cried a lot*, and *I was a bad person* over time. Age was also associated with lower subsequent levels of *I was no good anymore*,* I hated myself*, *nobody really loved me*, and *not as good as other kids.*

In males, age was associated with higher subsequent levels of *feeling miserable*, *did not enjoy anything*,* felt tired*,* I was a bad person*, and *felt lonely*. However, age was also associated with lower levels over time of *cried a lot*,* not as good as other kids*, and *did everything wrong*.

## Discussion

We examined how changes in pubertal status and chronological age influence individual depressive symptoms in adolescents, focusing on pathoplastic effects across sex. We investigated the influence of pubertal status on individual depressive symptoms from ages 10 to 18 in a large population-based study to capture within-person fluctuations.

Our findings are consistent with prior conclusions that pubertal status was associated with overall depression in females, but not males [11]. As found previously in females [29], advanced breast and pubic hair development were associated with higher levels of depression. Our within-person analyses reduce the likelihood of between-person confounding or reverse causation. Reverse causation is also offset by pubertal measures being reported the wave before depressive symptoms. Across models, we found some pathoplastic effects of pubertal status on individual depressive symptoms when accounting for overall depression severity and chronological age. Yet, more individual symptoms were associated with chronological age. Thus, our results support theories of a complex and sex-differentiated interaction between social and hormonal factors in adolescence which contributes to depressive symptoms [2, 4].

Chronological age showed relatively broad and consistent associations across depressive symptoms, whereas pubertal status indicators were more selectively linked to specific symptom expressions, particularly in females. This suggests partial convergence of findings in that both developmental processes contribute to changes in depressive symptomatology during adolescence, but also divergence in the specificity of effects across symptom domains. Specifically, age appeared to reflect more general developmental influences across affective, somatic, and self-evaluative symptoms, with increases (e.g., *felt tired*, *felt miserable*, *cried a lot*) and decreases (e.g., *not as good as other kids*, *I hated myself*) observed across sexes. In contrast, pubertal development showed more domain-specific associations, most prominently with self-evaluative symptoms in females and more limited effects on social and somatic symptoms in males. Convergence between age and pubertal effects was evident for some symptoms across sex, suggesting partially shared developmental influences.

Specifically, our findings align with prior work that self-esteem increases during adolescence [52], but also reveal that changes in pubertal status among females appear to increase negative self-evaluations. In both sexes, as adolescents aged, they reported lower levels of being *not as good as other kids*. Age was also inversely associated with *I was no good anymore* in females and with *did everything wrong* in males. However, in females, advancing changes in breast development were associated with higher levels of the symptoms *I hated myself* and *nobody really loved me.* At the same time, these symptoms had higher symptom levels in younger adolescents. The same pattern was found for pubic hair development and the symptom *I hated myself*. While self-esteem generally increases during adolescence [52], changes in pubertal status appear to interfere with these processes in females, potentially contributing to the emergence of gender differences in depression during puberty [1, 2]. Within-person changes of the symptom *I was a bad person* were associated with age in both sexes. This may be related to elevated conflicts with parents, teachers, or peers as adolescents seek autonomy.

Predictors of loneliness differed by sex. Of the pubertal indicators, changes in breast development were uniquely associated with lower levels of *felt lonely*, which may be attributable to more social aspects of this physical development. Visible physical changes may have attracted positive attention from peers and potential romantic interests, leading to increased social interactions. Or, females could receive more parental support with advancing pubertal status. Feelings of loneliness in males increased with age and greater voice changes, suggesting they received less social support or felt more distant from their peers. However, one could have expected different effects because voice deepening in males is often perceived as a sign of maturation and increased social status. Hence, further clarification of this finding is needed in future research.

*Feeling miserable* had different predictors by biological sex. Pubic hair development more strongly predicted this symptom in females and age more strongly predicted this in males. However, in univariate models all pubertal and age associations with this symptom were significant. During puberty, there are significant physiological and hormonal changes [4–6]. This can result in decreased mood owing to hormonal fluctuations (particularly in females), or an interaction with body image concerns or cultural and gender expectations [3, 5]. Age effects may be attributable to a range of different factors including increased social expectations or school demands [53].

There were opposite effects of age between sexes for the symptom *cried a lot*, with a positive association in females and a negative association in males. The latter may reflect social norms of emotional expression in men who are often socialized to be ‘strong’ and avoid showing vulnerability.

Of the physical symptoms of depression, only *felt tired* had significant predictors. Age was associated with higher levels of this symptom in both sexes, potentially reflecting increasing academic and social demands. Of the pubertal factors, only increased testes development was independently associated with higher levels of *felt tired.* Puberty can induce feelings of tiredness due to hormonal alterations impacting sleep patterns, augmented energy requirements from rapid growth and physical activities or emotional stress [4–6].

### Future research and potential clinical implications

Future research should examine the consistency of our findings in other samples and why pubertal indicators had distinct sex- or gender-differentiated effects on symptoms, in particular disentangling effects of hormonal (more sex-related factors), social (more gender-related factors), and cognitive change. In our study, we specifically focused on Tanner Stages and voice changes rather than other indicators due to the importance of these secondary sex characteristics for both pubertal development and depressive symptoms [12, 20]. This approach highlights the potential influence of biological (sex) and social (gender) factors. Thus, overall our research is helpful as a first step to guide future work. For now, our approach is not comprehensive, being based on available data in ALSPAC. Future studies should consider additional sex- and gender-specific factors that may contribute to depressive symptoms along with hormonal markers ([10, 11, 23]. For example, in females, aspects of the menstrual cycle could be examined alongside social influences. Furthermore, physical changes in height and weight could also serve as important variables, as they may exert individual pathoplastic effects or clarify the role of pathoplastic influences in the current study. Future studies may also investigate more specific time-varying pathoplastic associations across different developmental periods [4, 28]. In our study, we did not examine pubertal timing relative to peers (i.e., early or late maturation), as we focused on within-person fluctuations. Thus, we did not capture whether developing earlier or later than peers confers additional risk for specific depressive symptoms. Prior research suggests that pubertal development outside of normative timelines, particularly early maturation in females, is associated with increased risk for depressive symptoms [11]. Future research should integrate both approaches by combining within-person models of pubertal change with indices of pubertal timing (e.g., relative to age norms or sample distributions) to determine whether developmental deviations exert additional pathoplastic effects on depressive symptom profiles. The elucidation of pathoplastic effects and sex-specific associations between changes in pubertal status, chronological age, and depressive symptoms in adolescents may ultimately offer clinical insights and guide appropriate support strategies, in school settings or at home [53].

### Strengths & limitations

This is the first study to examine pathoplastic influences of pubertal status and chronological age on the manifestation of depression throughout adolescence. Pubertal indicators and age shaped individual depressive symptoms in different directions, and differently by sex, which helps clarify the complex relationship between puberty and depression. Additionally, this study takes a within-person approach to examine the relationship between pubertal status, age, and depression.

However, our focus primarily on secondary sex characteristics that differ in visibility between sexes may overlook important social dimensions of pubertal development. For instance, while breast development in female individuals is highly visible and known to impact social interactions, characteristics selected for analysis in male individuals, such as pubic hair and genital development, do not have the same visibility. Although we examined voice changes in male individuals, voice cracking can begin in different Tanner Stages, making comparison of voice cracking across participants of limited validity in assessing pubertal status [21]. While we tested for direct effects of pubertal status and age on depressive symptoms, these factors may interact, and other causal mechanisms may explain the observed effects. Further, time-lags between the measurement of puberty and depressive symptoms (ranging from 0.74 to 1.15 years) may not adequately capture fluctuations in mood and behaviour related to puberty. Results are specific to the ALSPAC sample with most participants having white ethnicity and too few non-white participants to consider moderation analyses. This is important as effects of puberty on depression in females seem to be more robust among white versus non-white females [11]. Likewise, depressive symptoms and puberty development are associated with socio-economic status (SES; [54, 55]); this moderator should be considered in future pathoplasty studies. We only investigated participants based on biological sex, as provided in ALSPAC, but acknowledge the need for this work to extend to gender non-conforming individuals [56]. There is much heterogeneity in depressive symptoms assessed across questionnaires, so results may not be extended to other scales [57]. While young people completed the depression measure, the primary caregiver assisted or completed the measure of pubertal development, which could introduce some confounding due to different reporters. Despite high correspondence among informants on the Tanner stages [43–45], older children have provided more accurate reports of their pubertal development than their mothers (females aged ≥ 11 years versus younger females; [46]). Moreover, the initial assessment of pubertal indicators only began at age 10, potentially missing earlier development before this age [42].

## Conclusion

Our study sheds light on the association of pubertal development and chronological age with subsequent individual depressive symptoms in adolescents. We found sex-specific patterns of pathoplastic effects from these predictors, highlighting the complexity of depressive symptom manifestation during adolescence.

## Supplementary Information

Below is the link to the electronic supplementary material.

Supplementary Material 1 (DOCX 23.3 KB)

## Data Availability

Data underlying the results presented in this paper are not publicly available. They can be requested from the ASLPAC study team by the means of a research proposal.

## Declarations

### AI Statement

During the preparation of this work the authors used ChatGPT in order to improve the clarity of the language. After using this tool/service, the authors reviewed and edited the content as needed and take full responsibility for the content of the published article.

## References

[1] Hyde JS, Mezulis AH, Abramson LY (2008) The ABCs of depression: Integrating affective, biological, and cognitive models to explain the emergence of the gender difference in depression. Psychol Rev 115(2):291–313. 10.1037/0033-295X.115.2.291 18426291

[2] Thapar A, Eyre O, Patel V, Brent D (2022) Depression in young people. Lancet 400(10352):617–631. 10.1016/S0140-6736(22)01012-1 35940184

[3] Toppari J, Juul A (2010) Trends in puberty timing in humans and environmental modifiers. Mol Cell Endocrinol Special Issue: Neuroendocrinol Puberty 324(1):39–44. 10.1016/j.mce.2010.03.011 20298746

[4] Dahl RE, Allen NB, Wilbrecht L, Suleiman AB (2018) Importance of investing in adolescence from a developmental science perspective. Nature 554(7693) Article 7693. 10.1038/nature25770 29469094

[5] Mendle J, Beltz AM, Carter R, Dorn LD (2019) Understanding Puberty and Its Measurement: Ideas for Research in a New Generation. J Res Adolescence 29(1):82–95. 10.1111/jora.12371 30869839

[6] Shirtcliff EA, Dahl RE, Pollak SD (2009) Pubertal Development: Correspondence Between Hormonal and Physical Development. Child Dev 80(2):327–337. 10.1111/j.1467-8624.2009.01263.x PMC2727719 19466995

[7] Cramer AO, Borsboom D, Aggen SH, Kendler KS (2012) The pathoplasticity of dysphoric episodes: Differential impact of stressful life events on the pattern of depressive symptom inter-correlations. Psychol Med 42(5):957–965 10.1017/S003329171100211X PMC3315770 22093641

[8] Klein DN, Kotov R, Bufferd SJ (2011) Personality and Depression: Explanatory Models and Review of the Evidence. Ann Rev Clin Psychol 7(1):269–295. 10.1146/annurev-clinpsy-032210-104540 PMC3518491 21166535

[9] Sawyer SM, Azzopardi PS, Wickremarathne D, Patton GC (2018) The age of adolescence. Lancet Child Adolesc Health 2(3):223–228. 10.1016/S2352-4642(18)30022-1 30169257

[10] Rudolph KD (2014) Puberty as a developmental context of risk for psychopathology. In *Handbook of developmental psychopathology, 3rd ed* (S. 331–354). Springer Science + Business Media. 10.1007/978-1-4614-9608-3_17

[11] Stumper A, Alloy LB (2023) Associations Between Pubertal Stage and Depression: A Systematic Review of the Literature. Child Psychiatry Hum Dev 54(2):312–339. 10.1007/s10578-021-01244-0 PMC11869324 34529199

[12] Marshall WA, Tanner JM (1968) Growth and Physiological Development During Adolescence. Annu Rev Med 19(1):283–300. 10.1146/annurev.me.19.020168.001435 4297619

[13] Grumbach MM (2004) The Neuroendocrinology of Human Puberty Revisited. Horm Res 57(Suppl):2–14. 10.1159/000058094 12065920

[14] Jones N-HY, Khoury JC, Xu Y, Newman N, Kalkwarf HJ, Braun JM, Lanphear B, Chen A, Cecil KM, Rose SR, Yolton K (2021) Comparing adolescent self staging of pubertal development with hormone biomarkers. J Pediatr Endocrinol Metab 34(12):1531–1541. 10.1515/jpem-2021-0366 PMC9195346 34432968

[15] Owens SA, Eisenlohr-Moul T (2018) Suicide Risk and the Menstrual Cycle: A Review of Candidate RDoC Mechanisms. Curr Psychiatry Rep 20(11):106. 10.1007/s11920-018-0962-3 PMC6844188 30293097

[16] Payne JL (2003) The role of estrogen in mood disorders in women. Int Rev Psychiatry 15(3):280–290. 10.1080/0954026031000136893 15276966

[17] Culbert KM, Milá Roa A, Stevens K, Sisk CL, Burt SA, Klump KL (2022) Pubertal emergence of testosterone effects on depressive symptoms in boys. JCPP Adv 2(3):e12088. 10.1002/jcv2.12088 PMC10195044 37206299

[18] Angold A (2003) Adolescent depression, cortisol and DHEA. Psychol Med 33(4):573–581. 10.1017/S003329170300775X 12785459

[19] Lin H-Y, Chen J-H, Chen K-H (2025) The Sex Hormone Precursors Dehydroepiandrosterone (DHEA) and Its Sulfate Ester Form (DHEAS): Molecular Mechanisms and Actions on Human Body. Int J Mol Sci 26(17):8568. 10.3390/ijms26178568 PMC12429729 40943486

[20] Dehestani N, Vijayakumar N, Ball G, Mansour L, Whittle S, S., Silk TJ (2023) Puberty age gap: New method of assessing pubertal timing and its association with mental health problems. Mol Psychiatry 1–8. 10.1038/s41380-023-02316-4 PMC11116096 38052980

[21] Vogt Yuan AS (2007) Gender Differences in the Relationship of Puberty with Adolescents’ Depressive Symptoms: Do Body Perceptions Matter? Sex Roles 57(1):69–80. 10.1007/s11199-007-9212-6

[22] Tarif D, Heron J, Fraser A, Elhakeem A, Joinson C (2025) Pubertal timing, depressive symptoms, and depression in adolescent males: A prospective cohort study. Psychol Med 55:e3. 10.1017/S0033291724003234 PMC11968118 39901618

[23] Ullsperger JM, Nikolas MA (2017) A meta-analytic review of the association between pubertal timing and psychopathology in adolescence: Are there sex differences in risk? Psychol Bull 143(9):903–938. 10.1037/bul0000106 28530427

[24] Wang H, Lin SL, Leung GM, Schooling CM (2016) Age at onset of puberty and adolescent depression:Children of 1997 birth cohort. Pediatrics 137(6). 10.1542/peds.2015-3231 27230766

[25] Jiang L, Hao Y, Wang Y, Chen Q, Xin G, Li P, Hui Y, Gao T, Li Q, Wang X (2025) Is early menarche related to depression? A meta-analysis. J Affect Disord 369:508–515. 10.1016/j.jad.2024.10.036 39393462

[26] Joinson C, Heron J, Lewis G, Croudace T, Araya R (2011) Timing of menarche and depressive symptoms in adolescent girls from a UK cohort. Br J Psychiatry 198(1):17–23. 10.1192/bjp.bp.110.080861 21200072

[27] Joinson C, Heron J, Araya R, Lewis G (2013) Early Menarche and Depressive Symptoms From Adolescence to Young Adulthood in a UK Cohort. J Am Acad Child Adolesc Psychiatry 52(6):591–598e2. 10.1016/j.jaac.2013.03.018 23702448

[28] Prince C, Joinson C, Kwong ASF, Fraser A, Heron J (2023) The relationship between timing of onset of menarche and depressive symptoms from adolescence to adulthood. Epidemiol Psychiatric Sci 32:e60. 10.1017/S2045796023000707 PMC10539742 37766510

[29] Joinson C, Heron J, Araya R, Paus T, Croudace T, Rubin C, Marcus M, Lewis G (2012) Association between pubertal development and depressive symptoms in girls from a UK cohort. Psychol Med 42(12):2579–2589. 10.1017/S003329171200061X 22717026

[30] Lewis G, Ioannidis K, van Harmelen A-L, Neufeld S, Stochl J, Lewis G, Jones PB, Goodyer I (2018) The association between pubertal status and depressive symptoms and diagnoses in adolescent females: A population-based cohort study. PLoS ONE 13(6):e0198804. 10.1371/journal.pone.0198804 PMC6005470 29912985

[31] Fried EI, Nesse RM, Zivin K, Guille C, Sen S (2014) Depression is more than the sum score of its parts: individual DSM symptoms have different risk factors. Psychol Med 44(10):2067–2076. 10.1017/S0033291713002900 PMC4104249 24289852

[32] Olino TM, Schlechter P, Klein DN, Kotov R, Seeley JR (2024) Personality and presentation of depression symptoms: a preliminary examination of the pathoplasticity model. Clin Psychol Sci. 10.1177/21677026241283401 PMC12306499 40735275

[33] Schlechter P, Rohde P, Seeley JR, Klein DN, Olino TM (2024) Examining the influence of episode number and age of onset on individual depressive symptoms across episodes of major depression. J Psychiatr Res 175:405–410. 10.1016/j.jpsychires.2024.05.039 38776861

[34] Moriarity DP, Slavich GM, Alloy LB, Olino TM (2023) Hierarchical inflammatory phenotypes of depression: a novel approach across five independent samples and 27,730 adults. Biol Psychiatry 93(3):253–259. 10.1016/j.biopsych.2022.08.017 PMC10014190 36802275

[35] Olino TM, Schlechter P, Grillo AR, Vrshek-Schallhorn S, Rohde P, Hayden EP, Seeley JR, Klein DN (2026) Perceived life events and presentation of depression symptoms: an examination of the pathoplasticity model. J Affect Disord 403:121489. 10.1016/j.jad.2026.121489 PMC13003349 41740750

[36] Barendse MEA, Byrne ML, Flournoy JC, McNeilly EA, Williamson G, Barrett V, Chavez A-MY, Shirtcliff SJ, Allen EA, N. B., Pfeifer JH (2022) Multimethod assessment of pubertal timing and associations with internalizing psychopathology in early adolescent girls. J Psychopathol Clin Sci 131(1):14–25. 10.1037/abn0000721 PMC9439585 34941314

[37] Copeland WE, Worthman C, Shanahan L, Costello EJ, Angold A (2019) Early pubertal timing and testosterone associated with higher levels of adolescent depression in girls. J Am Acad Child Adolesc Psychiatry 58(12):1197–1206. 10.1016/j.jaac.2019.02.007 PMC6693999 30768421

[38] Boyd A, Golding J, Macleod J, Lawlor D, Fraser A, Henderson J, Molloy L, Ness A, Ring S, Smith D, G (2012) Cohort profile: the ’children of the 90s’-the index offspring of the avon longitudinal study of parents and children. Int J Epidemiol 42. 10.1093/ije/dys064 PMC3600618 22507743

[39] Fraser A, Macdonald-Wallis C, Tilling K, Boyd A, Golding J, Davey Smith G, Henderson J, Macleod J, Molloy L, Ness A (2013) Cohort profile: the avon longitudinal study of parents and children: ALSPAC mothers cohort. Int J Epidemiol 42(1):97–110 10.1093/ije/dys066 PMC3600619 22507742

[40] Turner N, Joinson C, Peters TJ, Wiles N, Lewis G (2014) Validity of the short mood and feelings questionnaire in late adolescence. Psychol Assess 26:752–762. 10.1037/a0036572 24749755

[41] Schlechter P, Wilkinson PO, Ford TJ, Neufeld SA (2023) The short mood and feelings questionnaire from adolescence to emerging adulthood: measurement invariance across time and sex. Psychol Assess. 10.1037/pas0001222 36951690

[42] Golding J, Iles-Caven Y, Northstone K, Fraser A, Heron J (2023) Measures of puberty in the Avon Longitudinal Study of Parents and Children (ALSPAC) offspring cohort. Wellcome Open Res 8. https://www.ncbi.nlm.nih.gov/pmc/articles/PMC11075130/10.12688/wellcomeopenres.19793.2 PMC11075130 38716046

[43] Brooks-Gunn J, Warren MP, Rosso J, Gargiulo J (1987) Validity of self-report measures of girls’ pubertal status. Child Dev, 829–841 3608653

[44] Duke PM, Litt IF, Gross RT (1980) Adolescents’ self-assessment of sexual maturation. Pediatrics 66(6):918–920 7454482

[45] Morris NM, Udry JR (1980) Validation of a self-administered instrument to assess stage of adolescent development. J Youth Adolesc 9(3):271–280 10.1007/BF02088471 24318082

[46] Terry MB, Goldberg M, Schechter S, Houghton LC, White ML, O’Toole K, Chung WK, Daly MB, Keegan THM, Andrulis IL, Bradbury AR, Schwartz L, Knight JA, John EM, Buys SS (2016) Comparison of clinical, maternal, and self pubertal assessments: implications for health studies. Pediatrics 138(1):e20154571. 10.1542/peds.2015-4571 PMC4925080 27279647

[47] Tanner JM (1952) The assessment of growth and development in children. Arch Dis Child 27(131):10–33. 10.1136/adc.27.131.10 PMC1988469 14904111

[48] Yentes RD, Wilhelm F (2021) Careless: Procedure for computing indices of careless responding (Version 1.2. 1)[Computer software]

[49] Hallquist MN, Wiley JF (2018) *MplusAutomation*: an R package for facilitating large-scale latent variable analyses in M *plus*. Struct Equation Modeling: Multidisciplinary J 25(4):621–638. 10.1080/10705511.2017.1402334 PMC6075832 30083048

[50] Muthén LK, Muthén B (2017) Mplus user’s guide: statistical analysis with latent variables, user’s guide. Muthén & Muthén

[51] Benjamini Y, Hochberg Y (1995) Controlling the false discovery rate: a practical and powerful approach to multiple testing. J Royal Stat Soc Ser B (Methodological) 57(1):289–300

[52] Orth U, Robins RW (2014) The Development of Self-Esteem. Curr Dir Psychol Sci 23(5):381–387. 10.1177/0963721414547414

[53] Caldwell DM, Davies SR, Hetrick SE, Palmer JC, Caro P, López-López JA, Gunnell D, Kidger J, Thomas J, French C, Stockings E, Campbell R, Welton NJ (2019) School-based interventions to prevent anxiety and depression in children and young people: a systematic review and network meta-analysis. Lancet Psychiatry 6(12):1011–1020. 10.1016/S2215-0366(19)30403-1 PMC7029281 31734106

[54] Joinson C, Kounali D, Lewis G (2017) Family socioeconomic position in early life and onset of depressive symptoms and depression: a prospective cohort study. Soc Psychiatry Psychiatr Epidemiol 52(1):95–103. 10.1007/s00127-016-1308-2 PMC5226994 27837235

[55] Mendle J, Moore SR, Briley DA, Harden KP (2016) Puberty, socioeconomic status, and depression in girls: evidence for gene x environment Interactions. Clin Psychol science: J Association Psychol Sci 4(1):3–16. 10.1177/2167702614563598 PMC7450762 32864196

[56] Deardorff J, Hoyt LT, Carter R, Shirtcliff EA (2019) Next steps in puberty research: broadening the lens toward understudied populations. J Res Adolescence 29(1):133–154. 10.1111/jora.12402 PMC6827435 30869847

[57] Fried EI (2017) The 52 symptoms of major depression: Lack of content overlap among seven common depression scales. J Affect Disord 208:191–197. 10.1016/j.jad.2016.10.019 27792962

