# Supplementary material for: How changes in pubertal status and chronological age shape individual depressive symptoms by sex: testing a pathoplasty model
Source: Eur Child Adolesc Psychiatry. 2026 May 18;35(9):2893–902. doi: 10.1007/s00787-026-03065-9 (PMC13695686; doi:10.1007/s00787-026-03065-9)
Supplement: Supplementary file 1 [file 787_2026_3065_MOESM1_ESM.docx]

**Supplemental Material for:**

**How Changes in Pubertal Status and Chronological Age Shape Individual Depressive Symptoms: Testing a Pathoplasty Model**

| **Supplemental Table 1**  *Effects of the puberty indicators on the depressive symptoms while controlling for the latent depression trait* | | | | |  |
| --- | --- | --- | --- | --- | --- |
| Symptom/ Indicator | Breasts | Testes | Pubic hair (f) | Pubic hair (m) | Voice change |
| 1. Miserable/unhappy | 1.07 (1.02-1.13)* | 1.06 (1.01-1.12)* | 1.07 (1.02-1.12)*** | 1.07 (1.02-1.11)*** | 1.16 (1.06-1.26)*** |
| 2. Did not enjoy anything | 0.98 (0.92-1.05) | 1.05 (0.99-1.12) | 0.97 (0.92-1.03) | 1.06 (1.00-1.11)* | 0.98 (0.87-1.1) |
| 3. Felt tired | 1.15 (1.10-1.21)*** | 1.16 (1.11-1.22)*** | 1.13 (1.08-1.18)*** | 1.11 (1.07-1.16)*** | 1.18 (1.08-1.28)*** |
| 4. Restless | 0.96 (0.91-1.00) | 1.00 (0.96-1.05) | 0.97 (0.93-1.01) | 0.99 (0.96-1.03) | 1.01 (0.93-1.1) |
| 5. I was no good anymore | 0.92 (0.86-0.99)* | 0.92 (0.84-1.00) | 0.94 (0.88-0.99)* | 0.93 (0.87-1.00) | 0.89 (0.76-1.04) |
| 6. Cried a lot | 1.04 (0.98-1.11) | 0.78 (0.71-0.86)*** | 1.04 (0.99-1.09) | 0.80 (0.74-0.86)*** | 0.70 (0.59-0.83)*** |
| 7. Hard to concentrate | 1.03 (0.98-1.08) | 1.07 (1.02-1.12)* | 1.02 (0.98-1.07) | 1.04 (1.00-1.09)* | 1.10 (1.01-1.2)** |
| 8. I hated myself | 0.91 (0.85-0.98)* | 0.89 (0.81-0.99)* | 0.93 (0.87-0.99)* | 0.90 (0.83-0.98)* | 0.85 (0.72-1.01)* |
| 9. I was a bad person | 1.24 (1.16-1.33)*** | 1.10 (1.02-1.18)* | 1.22 (1.15-1.29)*** | 1.09 (1.03-1.16)*** | 1.14 (1.01-1.28)* |
| 10. Felt lonely | 0.93 (0.88-0.98)** | 1.09 (1.02-1.16)* | 0.96 (0.92-1.00) | 1.10 (1.05-1.16)*** | 1.28 (1.14-1.43)*** |
| 11. Nobody really loved me | 0.98 (0.91-1.05) | 1.06 (0.96-1.16) | 0.95 (0.90-1.01) | 1.06 (0.98-1.15) | 1.14 (0.96-1.35) |
| 12. Not as good as other kids | 0.86 (0.81-0.91)** | 0.84 (0.79-0.91)*** | 0.86 (0.82-0.91)*** | 0.85 (0.80-0.90)*** | 0.73 (0.65-0.83)*** |
| 13. Did everything wrong | 0.97 (0.91-1.04) | 0.98 (0.91-1.07) | 0.96 (0.91-1.02) | 0.97 (0.91-1.03) | 0.83 (0.72-0.95)*** |
| *Note.* Results are based on ordinal regression models and present odds ratios.  f = female, m = male  The number in the brackets refer to the 99% confidence intervals. ****p* < .001, ***p* < .01, **p* < .05  Note that these are Benjamini-Hochberg corrected p-values. | | | | |  |

| **Supplemental Table 2**  *Effects of age on the depressive symptoms while controlling for the latent depression trait* | | |
| --- | --- | --- |
| Symptom/ Indicator | Age (f) | Age (m) |
| 1. Miserable/unhappy | 1.07 (1.02-1.13)** | 1.1 (1.05-1.15)** |
| 2. Did not enjoy anything | 0.99 (0.93-1.05) | 1.07 (1.01-1.13)* |
| 3. Felt tired | 1.23 (1.17-1.29)*** | 1.16 (1.11-1.21)*** |
| 4. Restless | 0.97 (0.92-1.01) | 0.99 (0.94-1.03) |
| 5. I was no good anymore | 0.89 (0.83-0.96)*** | 0.91 (0.84-0.98)* |
| 6. Cried a lot | 1.08 (1.02-1.14)* | 0.76 (0.7-0.83)*** |
| 7. Hard to concentrate | 1.05 (1-1.1) | 1.06 (1.01-1.11)* |
| 8. I hated myself | 0.84 (0.78-0.9)*** | 0.87 (0.8-0.95)*** |
| 9. I was a bad person | 1.28 (1.2-1.36)*** | 1.11 (1.05-1.19)*** |
| 10. Felt lonely | 0.96 (0.91-1.01) | 1.14 (1.07-1.21)** |
| 11. Nobody really loved me | 0.9 (0.84-0.97)** | 1.08 (1.00-1.18) |
| 12. Not as good as other kids | 0.82 (0.78-0.87)*** | 0.83 (0.77-0.88)*** |
| 13. Did everything wrong | 0.98 (0.92-1.04) | 0.93 (0.87-1) |
| *Note.* Results are based on ordinal regression models and present odds ratios.  f = female, m = male  The number in the brackets refer to the 99% confidence intervals. ****p* < .001, ***p* < .01, **p* < .05  Note that these are Benjamini-Hochberg corrected p-values. | | |
